# Enhanced Electrochemical Properties of Zr^4+^-doped Li_1.20_[Mn_0.52_Ni_0.20_Co_0.08_]O_2_ Cathode Material for Lithium-ion Battery at Elevated Temperature

**DOI:** 10.1038/s41598-018-21345-6

**Published:** 2018-02-14

**Authors:** Yi Lu, Min Pang, Shiliang Shi, Qing Ye, Zhaojun Tian, Tao Wang

**Affiliations:** 1 Hunan University of Science and Technology, Work Safety Key Lab on Prevention and Control of Gas and Roof Disasters for Southern Coal Mines, Xiangtan, Hunan 411201 China; 2 Hunan University of Science and Technology, Hunan Province Key Laboratory of Safe Mining Techniques of Coal Mines, Xiangtan, Hunan 411201 China; 3 Hunan University of Science and Technology, School of Resource, Environment and Safety Engineering, Xiangtan, Hunan 411201 China

## Abstract

The typical co-precipitation method was adopted to synthesized the Li-excess Li_1.20_[Mn_0.52−*x*_Zr_*x*_Ni_0.20_Co_0.08_]O_2_ (*x* = 0, 0.01, 0.02, 0.03) series cathode materials. The influences of Zr^4+^ doping modification on the microstructure and micromorphology of Li_1.20_[Mn_0.52_Ni_0.20_Co_0.08_]O_2_ cathode materials were studied intensively by the combinations of XRD, SEM, LPS and XPS. Besides, after the doping modification with zirconium ions, Li_1.20_[Mn_0.52_Ni_0.20_Co_0.08_]O_2_ cathode demonstrated the lower cation mixing, superior cycling performance and higher rate capacities. Among the four cathode materials, the Li_1.20_[Mn_0.50_Zr_0.02_Ni_0.20_Co_0.08_]O_2_ exhibited the prime electrochemical properties with a capacity retention of 88.7% (201.0 mAh g^−1^) after 100 cycles at 45 °C and a discharge capacity of 114.7 mAh g^−1^ at 2 C rate. The EIS results showed that the Zr^4+^ doping modification can relieve the thickening of SEI films on the surface of cathode and accelerate the Li^+^ diffusion rate during the charge and discharge process.

## Introduction

Recently, the Li-excess Li_1.20_[Mn_0.52_Ni_0.20_Co_0.08_]O_2_ (0.6Li_2_MnO_3_·0.4LiNi_0.50_Co_0.20_Mn_0.30_O_2_) materials have attracted much study as cathodes for LIBs owing to the high specific discharge capacity (up to 250 mAh g^−1^) and less cost^1–3^. With further research, people have discovered that the Li_2_MnO_3_ phase (one of the components in the Li_1.20_[Mn_0.52_Ni_0.20_Co_0.08_]O_2_) will be activated and participate in the electrochemical reactions only when the cell voltage is charged to exceed 4.5 V^4,5^. However, the high working voltage will cause some drawbacks, such as severe capacity degradation and poor thermal stability, which restrict the practical applications, especially in EV and HEV^6,7^. Moreover, when the batteries have been used in the high temperature circumstance, the side reaction between the cathode and electrolyte will be more severe in comparison with the traditional cathode materials, such as LiCoO_2_ or LiNi_0.50_Co_0.20_Mn_0.30_O_2_ owing to the high working voltage for the Li-excess Li_1.20_[Mn_0.52_Ni_0.20_Co_0.08_]O_2_ materials^8^.

To maintain the stability of the cathode at high temperature, considerable effort has been made to resolve the intrinsic defects. For examples, the surface coating modification can effectively protect the cathode from reacting with the electrolyte and retard the thickening of SEI film during cycling. In addition, the suppression of the layered-to-spinel transformation for the Li-excess cathode materials can be obtained by the compact coating layer, leading to the improved electrochemical properties^9–12^. The ion doping modification can stabilize the cathode crystal structure and suppress the layer structural damage^13,14^. However, the surface coating modification technology has been complicated and the coating effect demonstrates to be difficult to control, while the ion doping modification shows the easy accessibility and obvious synthetic efficiency^15^. Therefore, the ion doping modification has been regarded as the competitive method to enhance the electrochemical properties of the Li-excess Li_1.20_[Mn_0.52_Ni_0.20_Co_0.08_]O_2_ materials.

Numerous studies have shown that the Zr^4+^ doping modification can effectively enhance the cyclical stability and rate capacity of cathodes. For example, when Zr^4+^ was doped into the LiCoO_2_ by using the ultrasonic spray pyrolysis method, the LiCo_0.99_Zr_0.01_O_2_ delivered the discharge capacity of 108 mAh g^−1^ at 1 C in the voltage range of 3.0–4.2 V after 50 cycles, while the un-doped sample rapidly dropped down to 23 mAh g^−1^ at the same condition^16^. When the LiNi_0.5_Co_0.2_Mn_0.3_O_2_ was doped modification with Zr^4+^ by solid-state method reaction, the Li(Ni_0.5_Co_0.2_Mn_0.3_)_0.09_Zr_0.01_O_2_ demonstrated the much more enhanced rate capability than that of the LiNi_0.5_Co_0.2_Mn_0.3_O_2_ by the suppression of electrode polarization^17^. While the radius of Zr^4+^ (0.072 nm) is larger than those of Mn^4+^ (0.053 nm), Ni^2+^ (0.069 nm), Co^3+^ (0.0685 nm) in the transition-metal layer, the Zr^4+^ adulteration will expand the diffusion path of Li^+^ insertion/extraction, leading to the improved electrochemical properties. On the other hand, the bond energy of Zr-O has found to be stronger than those of Ni-O Co-O and Mn-O, which will contribute to stabilizing the structure of cathode^18^. Based on the above evidence, Zr^4+^ will be an attractive doping element to dope into the Li-excess Li_1.20_[Mn_0.52_Ni_0.20_Co_0.08_]O_2_ and enhance the electrochemical properties.

In the work, the Li_1.20_[Mn_0.52−*x*_Zr_*x*_Ni_0.20_Co_0.08_]O_2_ (*x* = 0, 0.01, 0.02, 0.03) series samples have been synthesized via using carbonate co-precipitation method. And then the combination of microstructural, particle morphology and electrochemical properties has been surveyed to evaluate the influence of different Zr^4+^ doping contents into Li_1.20_[Mn_0.52_Ni_0.20_Co_0.08_]O_2_ cathode.

## Experimental

The Li-excess Li_1.20_[Mn_0.52−*x*_Zr_*x*_Ni_0.20_Co_0.08_]O_2_ (*x* = 0, 0.01, 0.02, 0.03) series cathode materials were synthesized via using the carbonate co-precipitation method to synthesize the carbonate precursors, followed by sintering with LiOH·H_2_O powder at high temperature to obtain the cathode materials. The typical synthesis route has been shown as follows: (1) The stoichiometric amounts of MnSO_4_·H_2_O, NiSO_4_·6H_2_O, CoSO_4_·7H_2_O and Zr(NO_3_)_4_·5H_2_O were dissolved in distilled water to obtain a transparent solution; (2) Then the appropriate amount of NH_3_·H_2_O, as chelating agent and Na_2_CO_3_, as precipitant, were dropped into the above solution to make the metal ions deposit uniformly; (3) The acquired [Mn_0.52−*x*_Zr_*x*_Ni_0.20_Co_0.08_](CO_3_)_0.80_ precursors were segregated, washed with deionized water until the impurities eliminate completely; (4) Then the stoichiometric amount of [Mn_0.52−*x*_Zr_*x*_Ni_0.20_Co_0.08_](CO_3_)_0.80_ precursors and an excess 3 wt.% amount of LiOH·H_2_O powder were mixed uniformly, followed by pre-heated at 500 °C for 6 h and finally calcined at 950 °C for 12 h in tube furnace to acquire the Li_1.20_[Mn_0.52−*x*_Zr_*x*_Ni_0.20_Co_0.08_]O_2_ (*x* = 0, 0.01, 0.02, 0.03) samples.

To investigate the influence of the Zr^4+^ doping on the crystal structure of Li_1.20_[Mn_0.52_Ni_0.20_Co_0.08_]O_2_, the XRD measurement were carried out by using Rigaku RINT2400 X-ray diffractometer with Cu K*α* radiation in the 10° ≤ 2*θ* ≤ 80°, accompanied by a step size of 0.02° and a count time of 10.0s. Rietveld refinement of the cathode powder diffraction patterns were performed by using the GSAS/EXPGUI program. The morphologies of Li_1.20_[Mn_0.52−*x*_Zr_*x*_Ni_0.20_Co_0.08_]O_2_ (*x* = 0, 0.01, 0.02, 0.03) were observed by using scanning electron microscopy (SEM, Ultra 55, Zeiss) and high-resolution transmission electron microscopy (TEM, FEI Titan G2 60–300) equipped with energy-dispersive X-ray spectroscopy (EDX, Oxford) to test the elemental distributions of cathode material (*x* = 0.02). The particle size was measured by using laser particle size Analysis (LPS, TOOLSO, 2005A). The chemical states of the doping element were determined by using X-ray photoelectron spectroscopy (XPS, Perkin Elmer PHI 1600). And the XPS spectra were fitted by using XPSPEAK software. The elemental composition, i.e. Ni, Co, Mn and Yb, was detected by ICP-OES (Inductively Coupled Plasma Optical Emission Spectrometer, iCAP 6000). Phase transformation studies of original and cycled Li_1.20_[Mn_0.52−*x*_Zr_*x*_Ni_0.20_Co_0.08_]O_2_ (*x* = 0, 0.02) were carried out using a micro-Raman spectrometer (LabRAMHREvolution, HORIBA).

The electrochemical properties of Li_1.20_[Mn_0.52−*x*_Zr_*x*_Ni_0.20_Co_0.08_]O_2_ (*x* = 0, 0.01, 0.02, 0.03) samples were measured by using galvanostatic charge and discharge with the coin cell of type CR2025. The coin cells were assembled as follows: (1) The 85 wt.% Li_1.20_[Mn_0.52−*x*_Zr_*x*_Ni_0.20_Co_0.08_]O_2_ (*x* = 0, 0.01, 0.02, 0.03) samples, 10 wt.% carbon black and 5 wt.% polyvinylidene fluoride were evenly mixed to form the cathode slurry; (2) Then the slurry was casted onto Al foil by using a smudge stick and dried at 110 °C for 12 h in vacuum drying oven, followed by squeezed and punched into a circular disc with *d* = 12 mm; (3) The as-prepared cathode plate, the lithium metal plate as anode, the Celgard 2400 as the separator and 1 M LiPF_6_ dissolved in EC/DMC at mass ratio of 1:1 as the electrolyte were assembled in an argon-filled glove box to form the coin cells. The Galvanostatic charge-discharge tests were carried out by on a Land CT2001A (Wuhan, China) tester.

The cells were charged and discharged in the voltage range of 2.0 to 4.8 V at the different current densities (1C = 250 mA g^−1^). In addition, the CHI660D workstation was used to perform the electrochemical impedance spectroscopy (a frequency range from 0.01 Hz to 100 kHz and perturbation amplitude of 5 mV) and the cyclic voltammogram (a voltage range from 2.0 V to 4.8 V with a scanning rate of 0.1 mV s^−1^).

## Results and Discussion

**Figure 1:**
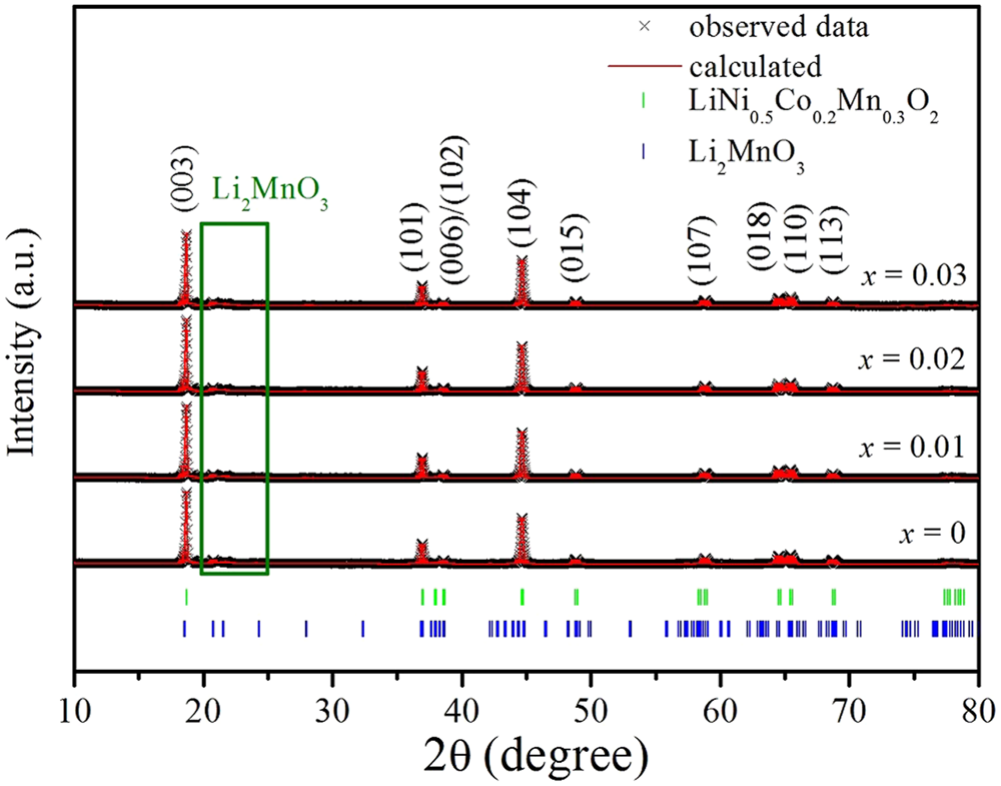
Profile fits for Rietveld refinement of the Li_1.20_[Mn_0.52−*x*_Zr_*x*_Ni_0.20_Co_0.08_]O_2_ (*x* = 0, 0.01, 0.02, 0.03) samples.

Figure 1 shows the X-ray diffraction patterns of the Li_1.20_[Mn_0.52−*x*_Zr_*x*_Ni_0.20_Co_0.08_]O_2_ (*x* = 0, 0.01, 0.02, 0.03)samples. The as-prepared samples have mainly demonstrated the typical XRD patterns of the hexagonal α-NaFeO_2_ structure with the space group R-3m (the LiMO_2_ features), except for the weak super lattice peaks between 20° and 25°, which are related to the Li_2_MnO_3_ phase, corresponding to the monocline unit cell C2/m^19,20^. In addition, the distinct splitting of (006)/(102) and (018)/(110) peaks have indicated that the as-prepared cathode materials have formed a well-developed hexagonal layered structure^21^. Besides, to further investigate the cation mixing between the Ni^2+^ and Li^+^ in the LiMO_2_ main phase, the Rietveld refinement of the diffraction patterns was performed based on the R-3m (used for LiNi_0.50_Co_0.20_Mn_0.30_O_2_ phase) and C2/m (used for Li_2_MnO_3_ phase) structure, as is shown in Fig. 1. And the structural parameters obtained from the refinement for the Li_1.20_[Mn_0.52−*x*_Zr_*x*_Ni_0.20_Co_0.08_]O_2_ (*x* = 0, 0.01, 0.02, 0.03) samples are listed in Table 1. It can be seen that with the Zr^4+^ doping content increasing, the lattice parameters *a* and *c* of LiNi_0.50_Co_0.20_Mn_0.30_O_2_ phase have gradually risen owing to the larger radius of Zr^4+^. The larger lattice parameters *a* and *c* will contribute to enhancing the Li^+^ diffusion rate during the charge and discharge process^22^. Besides, the *c*/*a* ratio is related to the cation mixing and a high ratio represents the well cation ordering has been formed^23^. It can be observed the Zr^4+^-doped samples deliver the higher *c*/*a* ratio than that of the un-doped cathode, indicating the cation mixing of the as-prepared samples has been improved after the Zr^4+^ doping. Besides, according to the reports of J.R. Dahn^24,25^, the nominal formula of Li_1.20_[Mn_0.52−*x*_Zr_*x*_Ni_0.20_Co_0.08_]O_2_ can be assumed as [Li_1−*δ*_Ni_*δ*_][Li_*δ*_Mn_0.52−*x*_Zr_*x*_Ni_0.20−*δ*_Co_0.08_]O_2_. The GSAS/EXPGUI program has been adopted to calculate the refined lattice structural data of as-prepared samples, as is shown in Table 1. It is clear that the amount of Ni in Li site for the Zr^4+^-doped samples is lower than that of the pristine Li_1.20_[Mn_0.52_Ni_0.20_Co_0.08_]O_2_ sample. And when the Zr^4+^ doping content aggrandizes, the amount of Ni in Li site first decreases from 0.059 to 0.041 and 0.032, then increases to 0.039, the Li_1.20_[Mn_0.50_Zr_0.02_Ni_0.20_Co_0.08_]O_2_ has demonstrated the optimal cation ordering. The lower cation mixing will not only suppress formation of spinel-like phase, but also improve the layered structure stability, finally contribute to enhancing the cyclic performance. Besides, the occupancy of Zr cations in 3*b*-site are respectively 0, 0.012, 0.019 and 0.031 with the Zr doping contents increasing, indicating the molar ratio for Zr doping can be designed experimentally. The Zr^4+^ doping can enlarge the lattice parameters, which facilitates Li-ion diffusion and subsequently enhances the high-rate capability.

**Table 1:** Structural parameters obtained from the refinement by the Rietveld method of the X-ray diffraction data recorded for the Li_1.20_[Mn_0.52−*x*_Zr_*x*_Ni_0.20_Co_0.08_]O_2_ (*x* = 0, 0.01, 0.02, 0.03) samples.

Sample	Lattice parameters						Cations occupancy (%)					Reliability factors		
	LiNi_0.50_Co_0.20_Mn_0.30_O_2_ phase			Li_2_MnO_3_ phase			*sit. Li/Ni occ*.	*3b Ni/Li occ*.	*3b Mn occ*.	*3b Zr occ*.	*3b Co occ*.	*R*_p_ (%)	*R*_wp_ (%)	*χ* ^2^
	*a*(Å)	*c*(Å)	*c/a*	*a(Å)*	*b(Å)*	*c(Å)*								
*x* = 0	2.8483 (2)	14.2154 (1)	4.9907	4.9812 (1)	8.5582 (1)	5.0542 (1)	1.141/0.059 (2)	0.141/0.059 (2)	0.518 (2)	0	0.082 (2)	7.88	9.16	1.59
*x* = 0.01	2.8515 (2)	14.2357 (2)	4.9925	4.9820 (2)	8.5588 (1)	5.0549 (1)	1.159/0.041 (3)	0.159/0.041 (2)	0.509 (3)	0.012 (3)	0.079 (2)	8.53	9.58	1.75
*x* = 0.02	2.8531 (1)	14.2469 (2)	4.9939	4.9825 (1)	8.5592 (2)	5.0552 (1)	1.168/0.032 (2)	0.168/0.032 (3)	0.500 (3)	0.019 (2)	0.081 (3)	8.21	9.62	1.69
*x = *0.03	2.8539 (1)	14.2522 (2)	4.9934	4.9818 (1)	8.5586 (2)	5.0547 (1)	1.161/0.039 (2)	0.161/0.039 (2)	0.491 (2)	0.031 (1)	0.078 (2)	7.32	8.93	1.50

**Figure 2:**
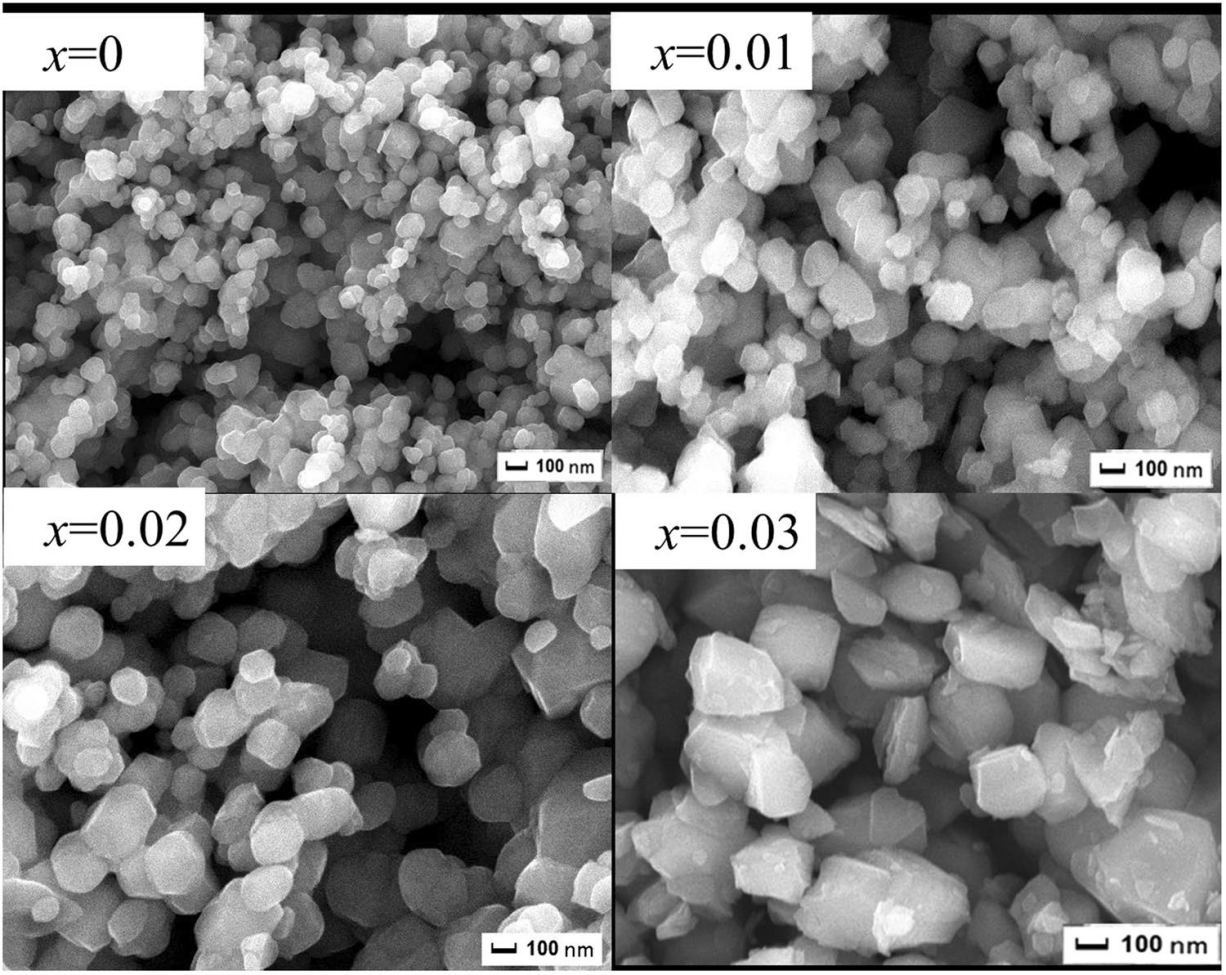
SEM images of the Li_1.20_[Mn_0.52−*x*_Zr_*x*_Ni_0.20_Co_0.08_]O_2_ (*x* = 0, 0.01, 0.02, 0.03) samples.

**Figure 3:**
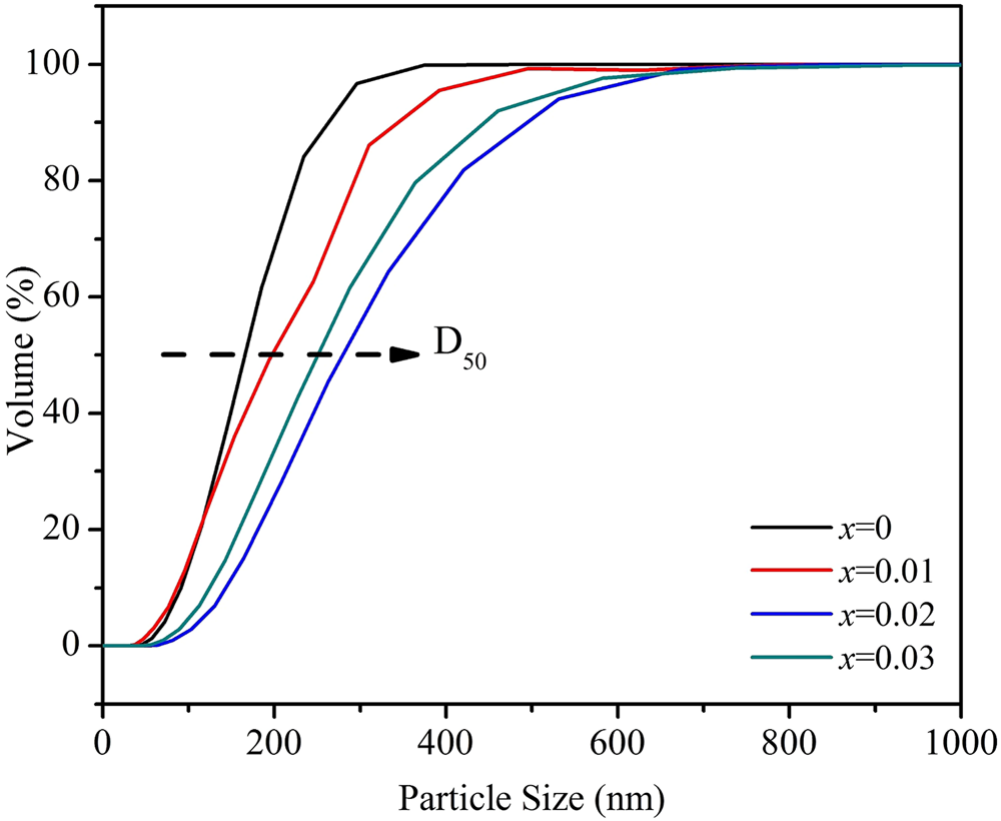
Size distribution of the Li_1.20_[Mn_0.52−*x*_Zr_*x*_Ni_0.20_Co_0.08_]O_2_ (*x* = 0, 0.01, 0.02, 0.03) samples.

**Figure 4:**
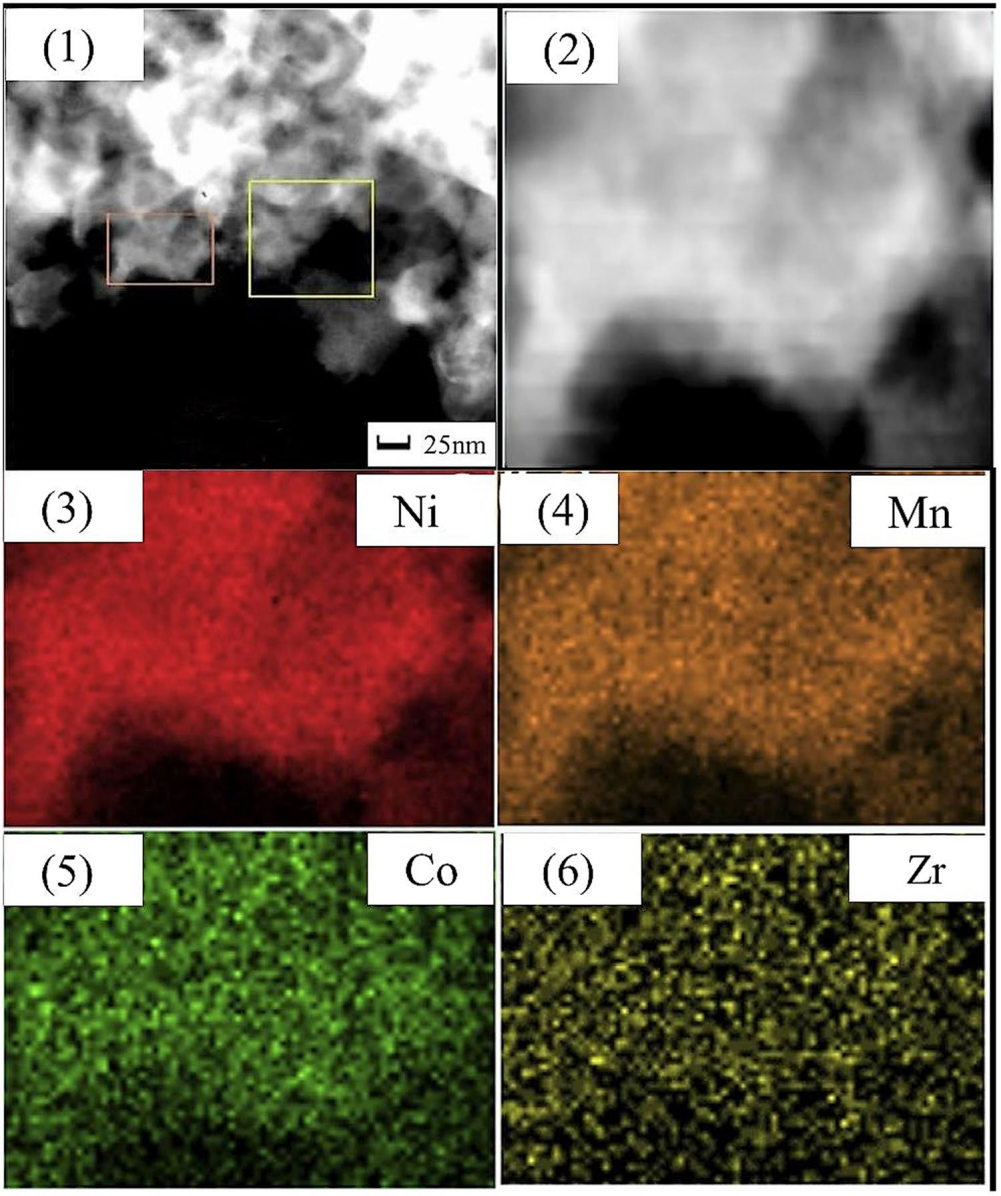
STEM images of Li_1.20_[Mn_0.50_Zr_0.02_Ni_0.20_Co_0.08_]O_2_ and corresponding elemental maps of Ni, Mn, Co and Zr.

Figure 2 shows the SEM images of the Li_1.20_[Mn_0.52−*x*_Zr_*x*_Ni_0.20_Co_0.08_]O_2_ (*x* = 0, 0.01, 0.02, 0.03) samples. The as-prepared samples are composed of numerous crystallites with a diameter of 200~700 nm. And all particles present the similar morphology of rock-shaped grains without obvious aggregation. In addition, with the Zr^4+^ doping content increasing, the crystal particles surface become more smooth and the size of the particles become larger, which implies the crystallinity of the particles can be enhanced after the Zr^4+^ doping. To further analyze the influence of the Zr^4+^ doping on the cathode particles size, the size distribution of the Li_1.20_[Mn_0.52−*x*_Zr_*x*_Ni_0.20_Co_0.08_]O_2_ (*x* = 0, 0.01, 0.02, 0.03) samples have been measured, as is shown in Fig. 3. It is obvious that the size of D_50_ gradually aggrandizes when the Zr^4+^ doping content increases, as is shown by the arrows, which is in good consistent with the observation of SEM images. A small amount of doped Zr ions may form continuous grain boundary phases in the Li_1.20_[Mn_0.52_Ni_0.20_Co_0.08_]O_2_ particles. These continuous grain boundary phases could enhance the mass diffusion transport at grain boundaries, finally promote the grain growth of Li_1.20_[Mn_0.52_Ni_0.20_Co_0.08_]O_2_^26,27^. And the well crystallization will help to ameliorate the electrochemical properties of cathode. Besides, the STEM images of Li_1.20_[Mn_0.50_Zr_0.02_Ni_0.20_Co_0.08_]O_2_ and corresponding elemental maps of Ni, Mn, Co and Zr is shown in Fig. 4. The Fig. 4 demonstrates that not only the Ni, Co and Mn atoms have been distributed homogeneously, but also the doping element Zr atom have been evenly distributed in the cathode particles rather than segregated on the oxide surface, indicating the Zr^4+^ doping technology has obtained the obvious synthetic efficiency. Based on the above analysis, it has proved that the Zr^4+^ has been successfully doped into the Li_1.20_[Mn_0.52_Ni_0.20_Co_0.08_]O_2_ cathode material with uniform dispersion. The uniform dispersion of Zr dopant will make the function of Zr^4+^ doping modification more stability, which may be ready to provide a better cycling performance to some extent.

**Figure 5:**
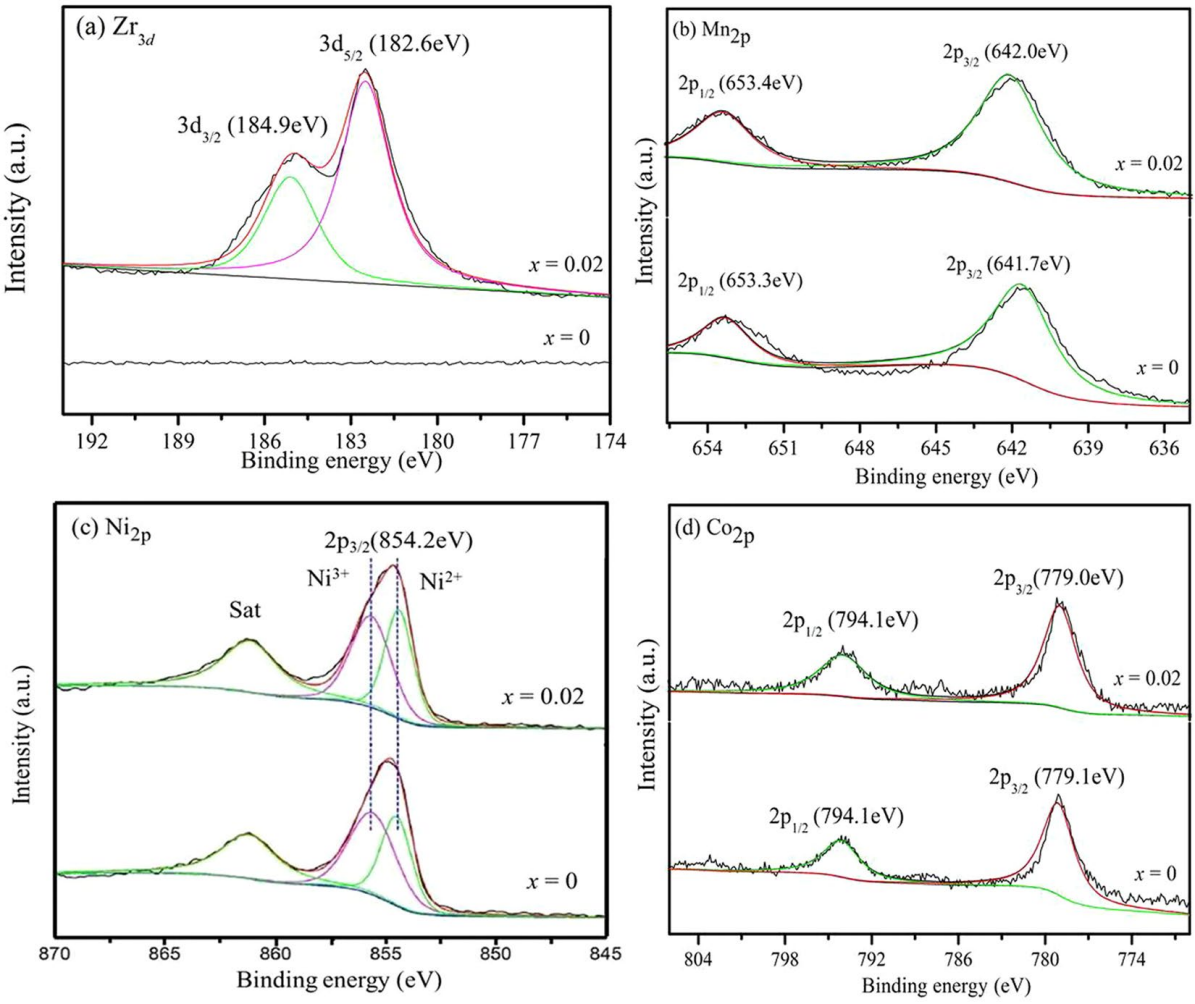
x-ray photoelectron spectroscopy (XPS) results of Zr, Mn, Ni and Co for the Li_1.20_[Mn_0.52−*x*_Zr_*x*_Ni_0.20_Co_0.08_]O_2_ (*x* = 0, 0.02) samples.

Figure 5 shows X-ray photoelectron spectroscopy (XPS) results of Zr, Mn, Ni and Co for the Li_1.20_[Mn_0.52−*x*_Zr_*x*_Ni_0.20_Co_0.08_]O_2_ (*x* = 0, 0.02) samples. In Fig. 5(a), the obvious peaks at the binding energies of 184.9 eV and 182.6 eV are assigned to Zr 3*d*_5/2_ and Zr 3*d*_3/2_, respectively, which corresponds to the Zr-O bonds at the state of Zr^4+ ^^28^. In Fig. 5(c), the obvious peaks at the binding energies of 854.2 eV is assigned to Ni_2*p*3/2_, which corresponds to the oxidation state of Ni^2+^ and Ni^3+^ after fitting, respectively^29,30^. Besides, it can be calculated that the relative content of Ni^2+^ decreased after zirconium doping owing to the reduction of cation mixing degree. Compared with the pristine Li_1.20_[Mn_0.52_Ni_0.20_Co_0.08_]O_2_, the binding energies of Mn_2p_ and Co_2p_ peaks for Li_1.20_[Mn_0.50_Zr_0.02_Ni_0.20_Co_0.08_]O_2_ have no obvious changes, indicating the chemical properties of the Mn and Co elements have not been changed after the Zr^4+^ doping modification.

To acquire the elements composition of Li_1.20_[Mn_0.52−*x*_Zr_*x*_Ni_0.20_Co_0.08_]O_2_ (*x* = 0, 0.01, 0.02, 0.03) samples, the ICP test was adopted and the results is demonstrated in Table 2. The measurement values of Ni, Co, Mn and Zr elements content are approximately equal to the theoretical analysis values, indicating that the molar ratios for Ni, Co, Mn and Zr elements have been synthesized in accordance with the experimental requirements.

**Table 2:** Relative contents of Ni, Co, Mn and Zr in the Li_1.20_[Mn_0.52−*x*_Zr_*x*_Ni_0.20_Co_0.08_]O_2_ (*x* = 0, 0.01, 0.02, 0.03) samples.

Sample	Theoretical molar ratio				Measurement molar ratio			
	Mn	Ni	Co	Zr	Mn	Ni	Co	Zr
*x = *0	0.540	0.130	0.130	0	0.542	0.129	0.129	0
*x = *0.01	0.530	0.130	0.130	0.010	0.533	0.129	0.128	0.010
*x = *0.02	0.520	0.130	0.130	0.020	0.518	0.131	0.130	0.021
*x = *0.03	0.510	0.130	0.130	0.030	0.512	0.128	0.131	0.029

**Figure 6:**
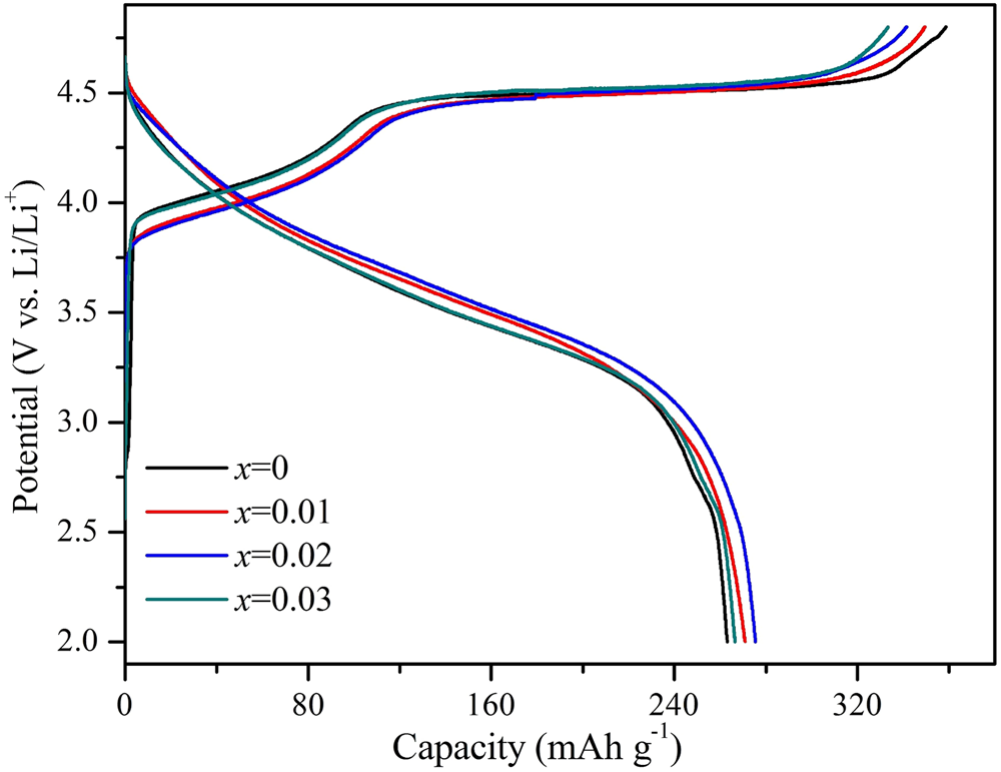
Initial charge-discharge curves of the Li_1.20_[Mn_0.52−*x*_Zr_*x*_Ni_0.20_Co_0.08_]O_2_ (*x* = 0, 0.01, 0.02, 0.03) samples in the voltage range of 2.0~4.8 V at 0.1 C rate.

Figure 6 shows the initial charge-discharge curves of the Li_1.20_[Mn_0.52−*x*_Zr_*x*_Ni_0.20_Co_0.08_]O_2_ (*x* = 0, 0.01, 0.02, 0.03) samples in the voltage range of 2.0~4.8 V at 0.1 C rate. All samples have demonstrated the similar charge curve for the two typical charge steps. The first step of charging process exists in the potential region from 2.0 V to 4.5 V, corresponding to the Li^+^-extraction from layer LiNi_0.50_Co_0.20_Mn_0.30_O_2_ component and the oxidation of Ni^2+^ to Ni^4+^ and Co^3+^ to Co^4+ ^^31,32^. For the second step, all samples exhibit a long voltage plateau at about 4.5 V, where the irreversible Li^+^ extract and oxygen release from the Li_2_MnO_3_ phase^33,34^. Table 3 shows the initial cycle electrochemical data of Li_1.20_[Mn_0.52−*x*_Zr_*x*_Ni_0.20_Co_0.08_]O_2_ (*x* = 0, 0.01, 0.02, 0.03) cathodes at 0.1 C rate in the voltage range of 2.0~4.8 V. With the Zr^4+^ doping content increasing, the initial charge capacities of as-prepared samples gradually decline owing to the electrochemical inactive of doped Zr^4+^, While the discharge capacities first enhance and then decrease and the Li_1.20_[Mn_0.50_Zr_0.02_Ni_0.20_Co_0.08_]O_2_ sample delivers the highest discharge capacity of 272.4 mAh g^−1^. In addition, the lowest irreversible capacity loss for the Li_1.20_[Mn_0.50_Zr_0.02_Ni_0.20_Co_0.08_]O_2_ sample has promoted the highest initial coulombic efficiency, which indicates that the Zr^4+^ doping can restrain the release of oxygen from the Li_2_MnO_3_ and decrease the irreversible capacity loss. Compared to the bonds break energy values for theΔ*H*ƒ_298_(Ni-O) = 391.6 kJ∙mol^−1^, Δ*H*ƒ_298_(Co-O) = 368 kJ∙mol^−1^ and Δ*H*ƒ_298_(Mn-O) = 402 kJ∙mol^−1^, the Zr-O delivers the higher bonds break energy value ofΔ*H*ƒ298(Zr-O) = 760 kJ mol^−1^, therefore with the Zr^4+^ doping, the oxygen release of the Zr^4+^-doped samples will face more resistance than the un-doped sample, subsequently the irreversible capacity loss has been suppressed^17^.

**Table 3:** Initial cycle electrochemical data of Li_1.20_[Mn_0.52−*x*_Zr_*x*_Ni_0.20_Co_0.08_]O_2_ (*x* = 0, 0.01, 0.02, 0.03) cathodes at 0.1 C rate in the voltage range of 2.0~4.8 V.

Sample	Charge capacity (mAh g^−1^)	Discharge capacity (mAh g^−1^)	Irreversible capacity loss (mAh g^−1^)	Coulombic efficiency (%)
0	356.4	263.5	92.9	73.9
0.01	349.9	270.5	79.4	77.3
0.02	344.5	272.4	72.1	79.1
0.03	336.2	266.7	69.5	79.3

**Figure 7:**
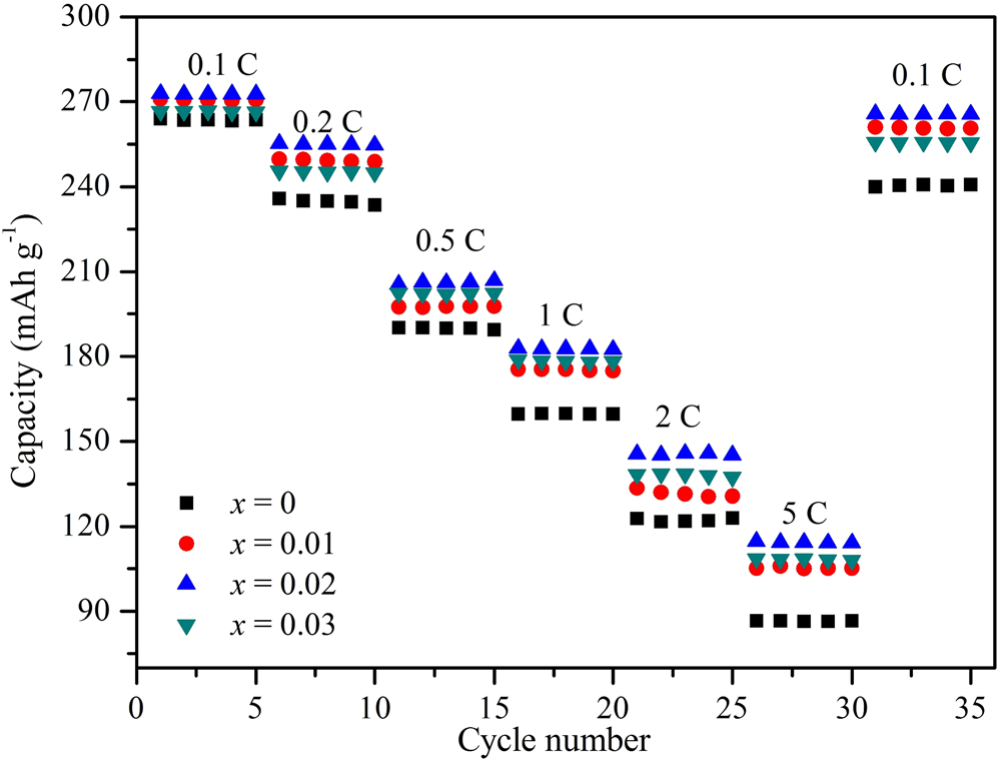
Rate capabilities of the Li_1.20_[Mn_0.52−*x*_Zr_*x*_Ni_0.20_Co_0.08_]O_2_ (*x* = 0, 0.01, 0.02, 0.03) samples with various current densities in the voltage range of 2.0~4.8 V.

Figure 7 shows the rate capabilities of the Li_1.20_[Mn_0.52−*x*_Zr_*x*_Ni_0.20_Co_0.08_]O_2_ (*x* = 0, 0.01, 0.02, 0.03) samples with various current densities in the voltage range of 2.0~4.8 V. Obviously, the Li_1.20_[Mn_0.52−*x*_Zr_*x*_Ni_0.20_Co_0.08_]O_2_ (*x* = 0.01, 0.02, 0.03) samples have all demonstrated the higher discharge capacities than those of the pristine Li_1.20_[Mn_0.52_Ni_0.20_Co_0.08_]O_2_ at the rate of 0.1 C, 0.2 C, 0.5 C, 1 C, 2 C and 5 C, thereinto the Li_1.20_[Mn_0.50_Zr_0.02_Ni_0.20_Co_0.08_]O_2_ sample delivers the optimum rate capacity. In addition, with current density increasing, the superiority has become particularly evident, indicating the advantage of Zr^4+^ doping on the rate capacity of Li_1.20_[Mn_0.52_Ni_0.20_Co_0.08_]O_2_ is much more significant at high rate. As is seen in Table 4, the discharge capacity of the Li_1.20_[Mn_0.50_Zr_0.02_Ni_0.20_Co_0.08_]O_2_ is only 8.8 mAh g^−1^ higher than that of the bare Li_1.20_[Mn_0.52_Ni_0.20_Co_0.08_]O_2_. However when the current density enhances to 5 C rate, the bare sample shows a discharge capacity of 86.6 mAh g^−1^ and this value is increased to 105.3, 114.7 and 108.6 mAh g^−1^ for the Li_1.20_[Mn_0.52−*x*_Zr_*x*_Ni_0.20_Co_0.08_]O_2_ (*x* = 0.01, 0.02, 0.03) samples, respectively. The superior rate capacity of the Zr^4+^-doped Li_1.20_[Mn_0.52_Ni_0.20_Co_0.08_]O_2_ samples have mainly been attributed to the fast Li^+^ migration speed during the charge and discharge process. One reason is that with the Zr^4+^ doping, the larger lattice parameters of the Zr^4+^-doped samples have contributed to enhancing the Li^+^ diffusion speed. Besides, the better crystallization property of the Zr^4+^-doped samples will also help to strengthen the conductivity ability of ions and electrons during the charge-discharge process.

**Table 4:** Discharge capacity of Li_1.20_[Mn_0.52−*x*_Zr_*x*_Ni_0.20_Co_0.08_]O_2_ (*x* = 0, 0.01, 0.02, 0.03) at various current densities in the voltage range of 2.0~4.8 V.

Sample	0.1 C rate (mAh g^−1^)	0.2 C rate (mAh g^−1^)	0.5 C rate (mAh g^−1^)	1 C rate (mAh g^−1^)	2 C rate (mAh g^−1^)	5 C rate (mAhg^−1^)	follow-up 0.1 C rate (mAh g^−1^)
*x = *0	264.0	235.8	190.2	159.6	122.8	86.6	240.0
*x = *0.01	271.3	249.7	197.6	175.4	133.5	105.3	261.0
*x = *0.02	272.8	255.1	205.5	182.8	145.6	114.7	265.8
*x = *0.03	266.8	245.5	202.4	178.8	138.4	108.6	255.7

**Figure 8:**
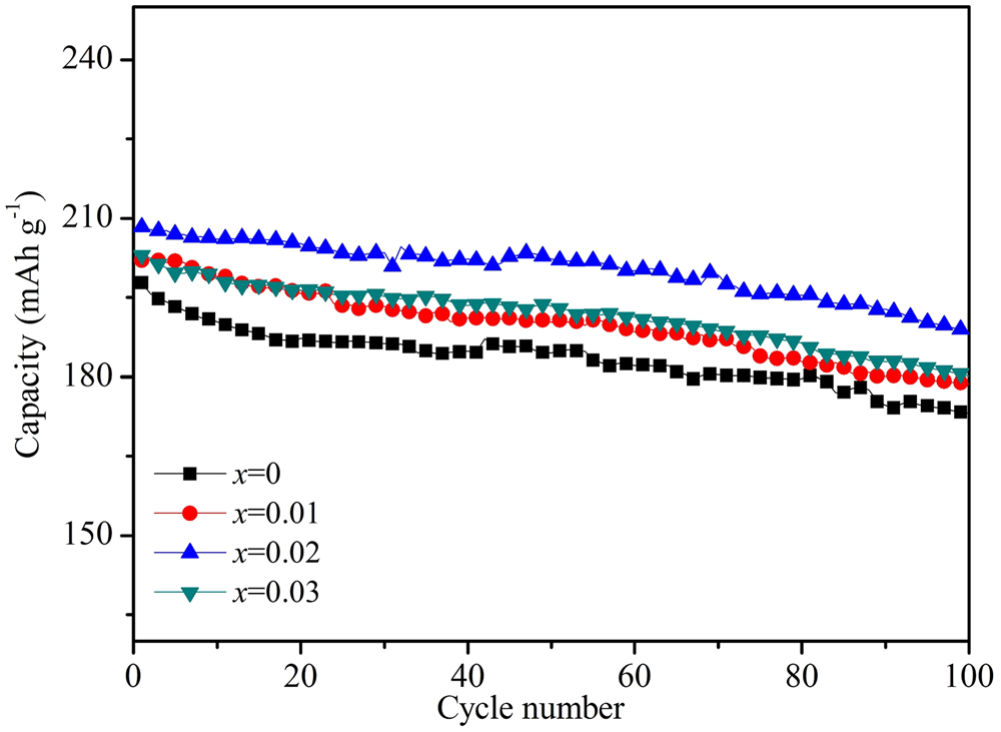
Cycling performance of the Li_1.20_[Mn_0.52−*x*_Zr_*x*_Ni_0.20_Co_0.08_]O_2_ (*x* = 0, 0.01, 0.02, 0.03) samples at 0.5 C rate in the voltage range of 2.0~4.8 V at 25 °C.

**Figure 9:**
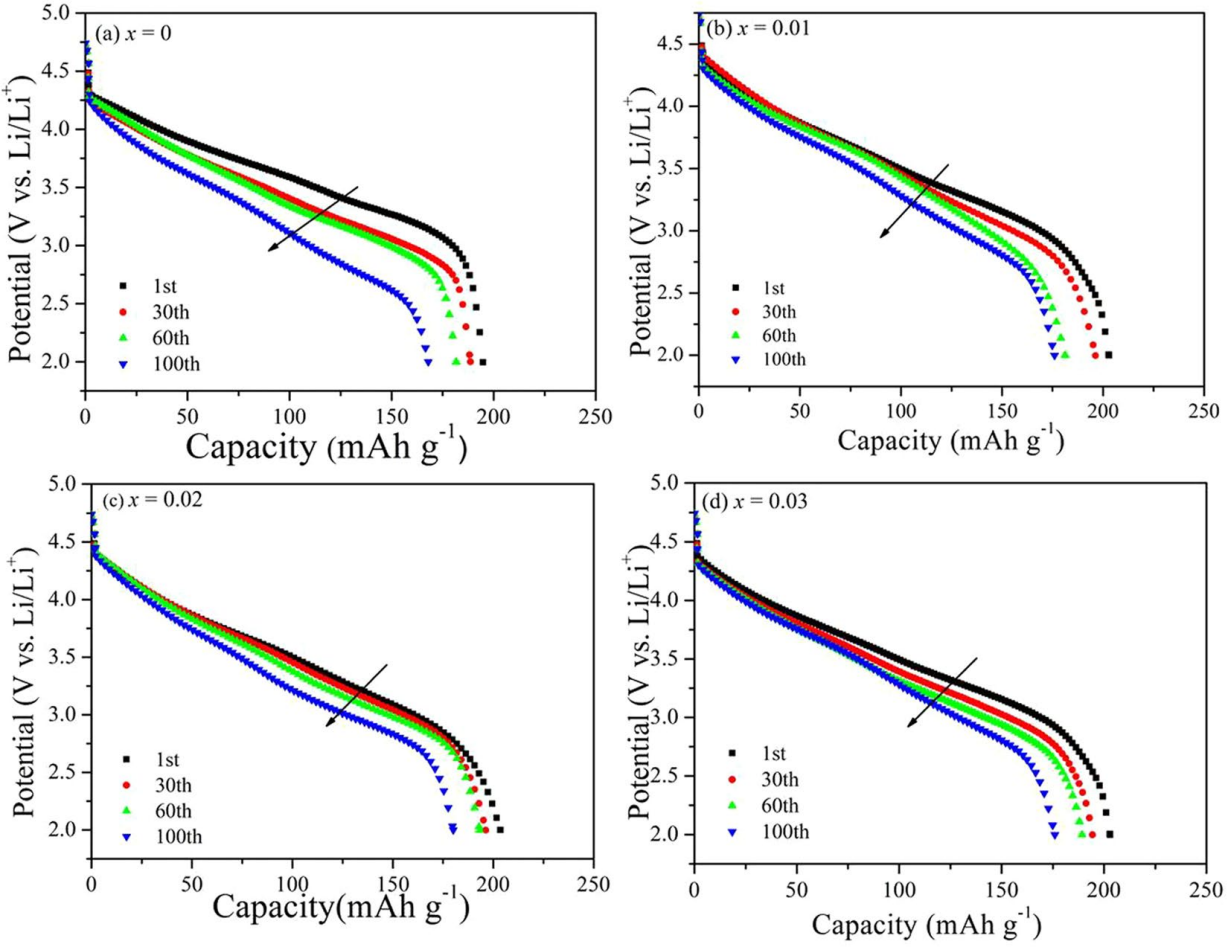
Discharge profiles of the Li_1.20_[Mn_0.52−*x*_Zr_*x*_Ni_0.20_Co_0.08_]O_2_ (*x* = 0, 0.01, 0.02, 0.03) samples from 2.0 V to 4.8 V at 0.5 C rate in the 1st, 30th, 60th and 100th cycles.

Figure 8 shows the cycling performance of the Li_1.20_[Mn_0.52−*x*_Zr_*x*_Ni_0.20_Co_0.08_]O_2_ (*x* = 0, 0.01, 0.02, 0.03) samples at 0.5 C rate in the voltage range of 2.0~4.8 V at room temperature (25 °C). It can be observed that the Zr^4+^-doped samples have delivered the higher discharge capacity than that of the bare Li_1.20_[Mn_0.52_Ni_0.20_Co_0.08_]O_2_. And with the cycles going on, the cycling performance of the bare Li_1.20_[Mn_0.52_Ni_0.20_Co_0.08_]O_2_ is similar to those of the Zr^4+^-doped samples, the discharge capacities have all gradually attenuated followed the same trend. Table 5 shows the discharge capacity of Li_1.20_[Mn_0.52−*x*_Zr_*x*_Ni_0.20_Co_0.08_]O_2_ (*x* = 0, 0.01, 0.02, 0.03) at 0.5 C rate in the voltage range of 2.0~4.8 V at 25 °C. With the Zr^4+^ doping content increasing, the initial discharge capacities are198.0, 202.0, 208.3 and 203.0 mAh g^−1^, respectively. And after 100 cycles, the corresponding capacity retentions still maintain 86.9%, 88.5%, 90.4% and 88.7%, respectively. It has proved that the Zr^4+^ doping modification can enhance the specific capacity and cycling performance of the Li_1.20_[Mn_0.52_Ni_0.20_Co_0.08_]O_2_ cathode, owing to the lower cation mixing and faster Li^+^ migration speed for the Zr^4+^-doped samples. Besides, the discharge voltage plateau will gradually decrease during the cyclic process, owing to the enlargement of polarization and the formation of spinel-like phase for cathode materials^35^. It can be observed that the discharge voltage drops to lower plateau for the all cathodes after different cycles, as the arrows pointed in Fig. 9. Table 5 shows the declining value of voltage plateau between 1st and 100th (ΔV) for the Li_1.20_[Mn_0.52−*x*_Zr_*x*_Ni_0.20_Co_0.08_]O_2_ (*x* = 0, 0.01, 0.02, 0.03) samples and with the Zr^4+^ doping contents increasing, the ΔV values are 0.298, 0.259, 0.211 and 0.236 V, respectively. The smaller ΔV values of the Zr^4+^-doped cathodes have indicated that the Zr^4+^ doping modification can improve the layered structural stability by restraining the cation mixing between the Ni^2+^ and Li^+^ and the formation of spinel-like phase. While the smaller ΔV values of Zr^4+^-doped cathodes will contribute to maintaining the high power output of cells.

**Table 5:** Discharge capacity and the difference value of discharge mid-point voltage (∆V) of Li_1.20_[Mn_0.52−*x*_Zr_*x*_Ni_0.20_Co_0.08_]O_2_ (*x* = 0, 0.01, 0.02, 0.03) at 0.5 C rate in the voltage range of 2.0–4.8 V at 25 °C.

Sample	Initial discharge specific capacity (mAh g^−1^)	100th Specific discharge capacity (mAh g^−1′^)	100 cycles capacity retention (%)	Declining value of voltage plateau (∆V) (V)
*x = *0	198.0	172.1	86.9	0.298
*x = *0.01	202.0	178.7	88.5	0.259
*x = *0.02	208.3	188.2	90.4	0.211
*x = *0.03	203.0	180.1	88.7	0.236

**Figure 10:**
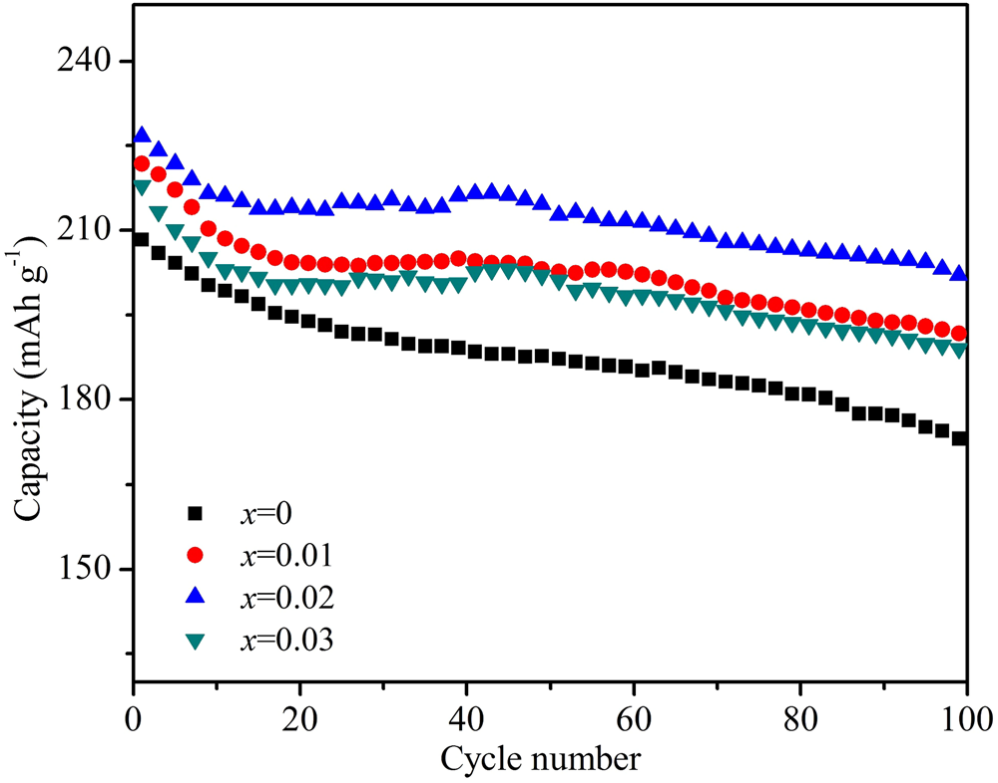
Cycling performance of the Li_1.20_[Mn_0.52−*x*_Zr_*x*_Ni_0.20_Co_0.08_]O_2_ (*x* = 0, 0.01, 0.02, 0.03) at 0.5 C rate in the voltage range of 2.0~4.8 V at 45 °C.

The poor cycling performance at high temperature for the Li-excess Li_1.20_[Mn_0.52_Ni_0.20_Co_0.08_]O_2_ has become one of the main drawbacks for the commercial application owing to the enhancement of the side reaction between cathode and electrolyte. Figure 10 shows the cycling performance of the Li_1.20_[Mn_0.52−*x*_Zr_*x*_Ni_0.20_Co_0.08_]O_2_ (*x* = 0, 0.01, 0.02, 0.03) at 0.5 C rate in the voltage range of 2.0~4.8 V at 45 °C. In comparison with the cycling performance at room temperature, the bare Li_1.20_[Mn_0.52_Ni_0.20_Co_0.08_]O_2_ demonstrates the more severe capacity fading. During the early cycle period, the fast capacity attenuation can be observed owing to the bare cathode particles surface. After several cycles, the side reaction between the cathode and electrolyte can generate some by-product, which will deposit at the electrode/electrolyte interface to form the Solid Electrolyte Interface (SEI) film. And the SEI film will protect the cathode materials from erosion by the electrolyte, making the capacity attenuation slightly slow^36,37^. The initial discharge capacities are 221.8, 226.6 and 218.0 mAh g^−1^ for Zr^4+^-doped Li_1.20_[Mn_0.52_Ni_0.20_Co_0.08_]O_2_ electrodes with the doping contents of 0.01, 0.02 and 0.03, respectively, larger than that (208.3 mAh g^−1^) of the un-doped Li_1.20_[Mn_0.52_Ni_0.20_Co_0.08_]O_2_, as is seen in Table 6. After 100 cycles, with the Zr^4+^ doping contents increasing, the Zr^4+^-doped Li_1.20_[Mn_0.52_Ni_0.20_Co_0.08_]O_2_ samples exhibit the discharge capacity of 180.2, 190.7 and 176.9 mAh g^−1^ respectively, corresponding that the capacity retentions first enhance from 86.3% to 88.7% and then decline to 86.5%. As for the bare Li_1.20_[Mn_0.52_Ni_0.20_Co_0.08_]O_2_, the discharge capacity decreases acutely to 172.5 mAh g^−1^ with the capacity retention of only 82.8%. During the charge-discharge process at high temperature, the cathodes have suffered from the attack of HF, dissolution of the Mn ions, structural change and decomposition of electrolyte on the cathode surface^38^. While the stronger total metal–oxygen bonding for the Zr^4+^-doped samples can contribute to stabilizing the structure of cathode during cycling, leading to the improved cycling performance. However, when the Zr^4+^ doping content reaches to 0.03, the cycling performance of Li_1.20_[Mn_0.49_Zr_0.03_Ni_0.20_Co_0.08_]O_2_ is not as good as that of the Li_1.20_[Mn_0.50_Zr_0.02_Ni_0.20_Co_0.08_]O_2_ for that the inhomogeneity phase of the ZrO_2_ existed in the compound can hinder the Li^+^ intercalation/deintercalation from the cathode^17^.

**Table 6:** Capacity retention and discharge capacity of Li_1.20_[Mn_0.52−*x*_Zr_*x*_Ni_0.20_Co_0.08_]O_2_ (*x* = 0, 0.01, 0.02, 0.03) at 0.5 C rate in the voltage range of 2.0–4.8 V at 45 °C.

Sample	Initial discharge specific Capacity (mAh g^−1^)	100th Specific discharge capacity (mAh g^−1′^)	100 cycles capacity retention (%)
*x = *0	208.3	172.5	82.8
*x = *0.01	221.8	191.4	86.3
*x = *0.02	226.6	201.0	88.7
*x = *0.03	218.0	188.5	86.5

**Figure 11:**
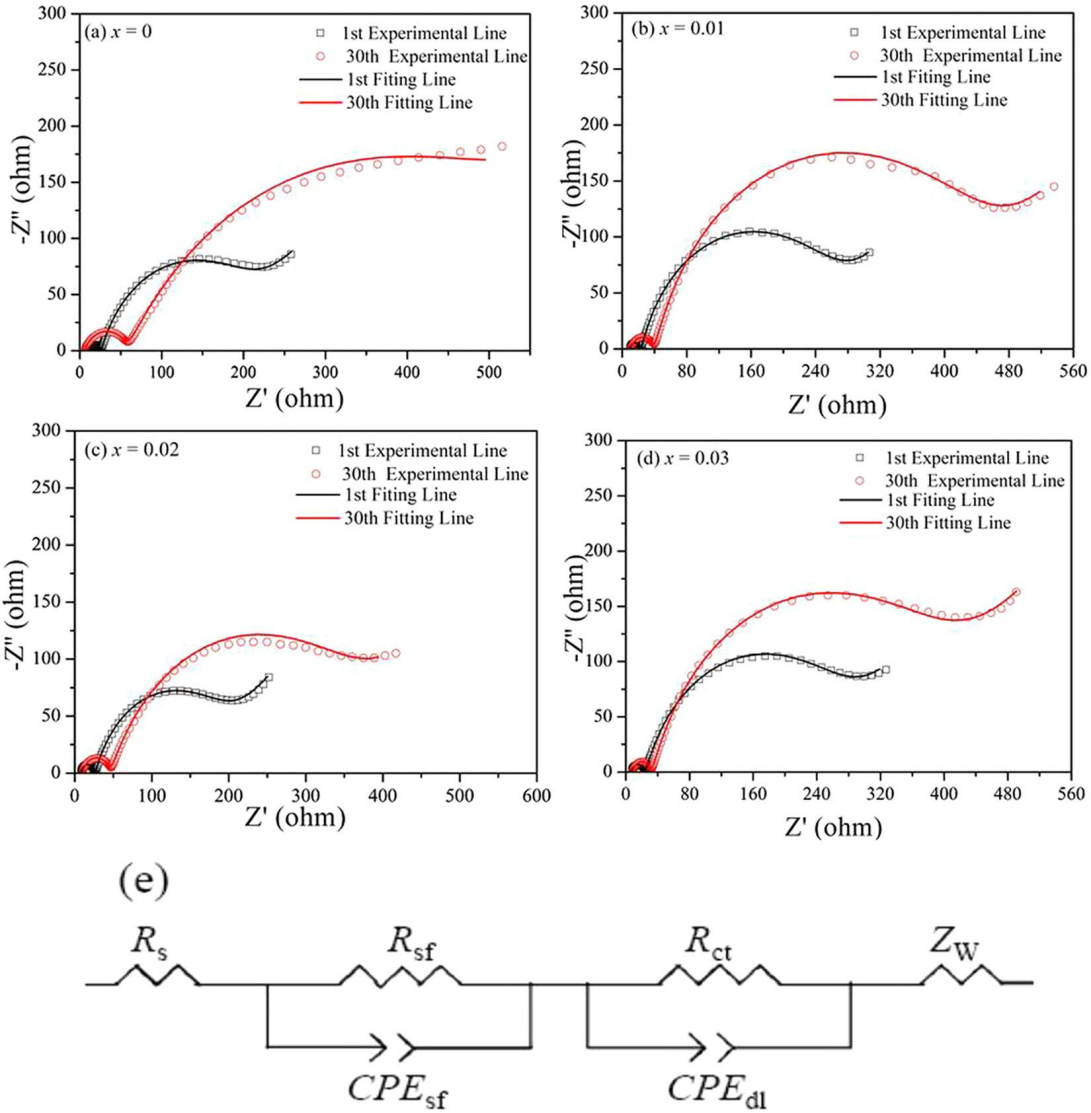
Nyquist plots of the Li_1.20_[Mn_0.52−*x*_Zr_*x*_Ni_0.20_Co_0.08_]O_2_ (*x* = 0, 0.01, 0.02, 0.03) samples at a charge state of 4.5 V in the 1st, 30th cycles and (**e**) the equivalent circuit used to fit the measured impedance spectra.

**Figure 12:**
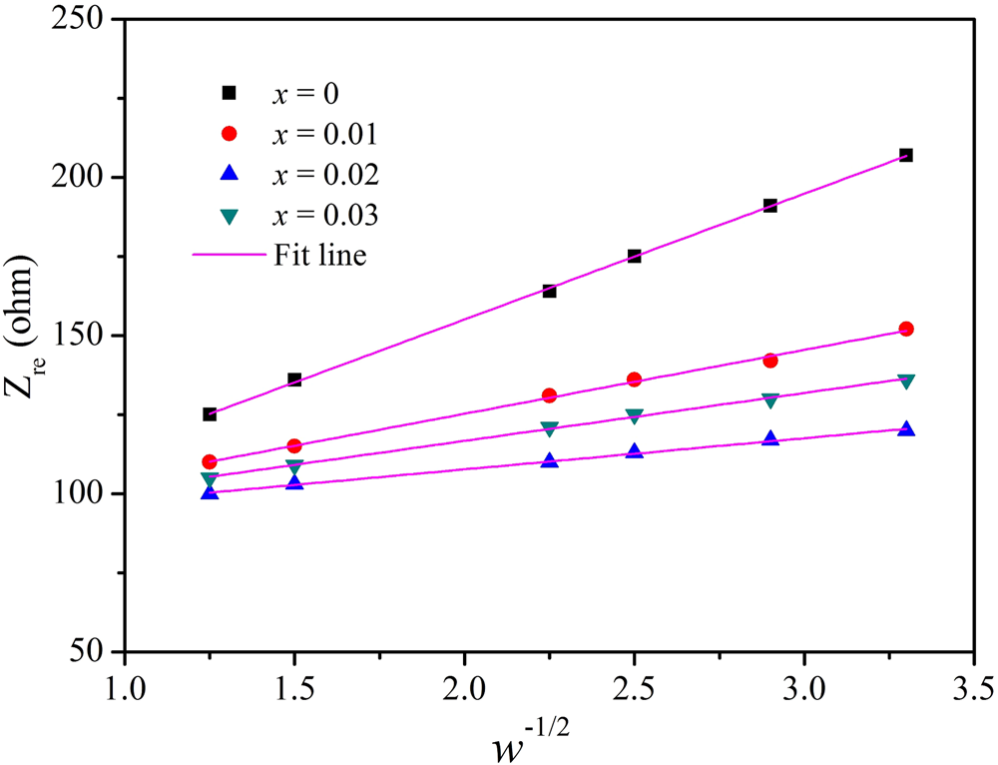
Plots comparison of Z_re_ *vs. ω*^−1/2^ for the Li_1.20_[Mn_0.52−*x*_Zr_*x*_Ni_0.20_Co_0.08_]O_2_ (*x* = 0, 0.01, 0.02, 0.03) samples after 30 cycles.

To further understand the influence of Zr^4+^ doping on the electrochemical properties of Zr^4+^-doped Li_1.20_[Mn_0.52_Ni_0.20_Co_0.08_]O_2_, the electrochemical impedance spectroscopy (EIS) for the four samples have been carried out after charging to 4.5 V in the 1st, 30th cycles. Figure 11 shows the Nyquist curves of the four cathodes and all the Nyquist curves demonstrate the similar characteristics, containing a small semicircle in the high frequency, a large semicircle in the high to medium frequency and a quasi-straight line in the low frequency, which respectively correspond to the impedance of Li^+^ migration across the SEI film (*R*_*sf*_ *and CPE*_*sf*_), the impedance of charge transfer (*R*_*ct*_ *and CPE*_*dl*_) and the impedance of Li-ion migration in the cathode (Z_*W*_)^39,40^. The corresponding equivalent circuit in Fig. 10(e) is used to simulate the Nyquist curves and the corresponding *R*_s_, *R*_sf_ and *R*_ct_ values can be acquired, as is shown in Table 7. In the 1st cycle, the *R*_*sf*_ values of Zr^4+^-doped Li_1.20_[Mn_0.52_Ni_0.20_Co_0.08_]O_2_ are lower than that of the bare one, therefore the superior initial discharge capacity can be obtained for the Zr^4+^-doped Li_1.20_[Mn_0.52_Ni_0.20_Co_0.08_]O_2_, which is in consistency with the results of Table 3. With the cycles going on, the SEI film will thicken, causing the increase of the *R*_*sf*_ value. After 30 cycles, with the Zr^4+^ doping contents increasing, the Zr^4+^-doped Li_1.20_[Mn_0.52_Ni_0.20_Co_0.08_]O_2_ samples deliver the *R*_*sf*_ values of 445.8, 363.1 and 428.8 Ω respectively, corresponding that the Δ*R*_*sf*_ values first enhance from 287.1 to 210.8 and then drop to 284.9 Ω. As for the bare Li_1.20_[Mn_0.52_Ni_0.20_Co_0.08_]O_2_, the *R*_*sf*_ value rise promptly to 544.5 Ω, with the Δ*R*_*sf*_ value of 371.4 Ω. It indicates the samples after the Zr^4+^ doping can relieve the thickening of SEI films on the surface of cathode, which contributes to decreasing the Li^+^ migration resistance across the SEI films and enhancing the electrochemical properties. Besides, the Li^+^ diffusion rate (*D*_*Li*_^+^) in the cathode can be calculated using the following equations^41^:

1 $${D}_{L{i}^{+}}=\frac{{R}^{2}{T}^{2}}{2{F}^{4}{n}^{4}{A}^{2}{C}^{2}{\tau }_{W}}$$

2 $${Z}_{re}={R}_{S}+{R}_{ct}+{\tau }_{W}{\omega }^{-1/2}$$

where, *F*, *n*, *A*, *C R* is gas constant, *T* is the absolute temperature, *F* represents the Faraday constant, *n* is the number of electrons per molecule during oxidation, *A* corresponds to the area of the electrode-electrolyte interface, i.e. 1.13 cm^2^ and *C* is the concentration of lithium ion, respectively. Besides, *τ*_*W*_ is the Warburg coefficient of the bulk cathode, which is can be calculated by the Eqs (2). Thereinto, the *Z*_re_ is the real part of impedance, ω is the angular frequency^42^ and Fig. 12 shows the plots comparison of Z_re_ *vs. ω*^−1/2^ for the Li_1.20_[Mn_0.52−*x*_Zr_*x*_Ni_0.20_Co_0.08_]O_2_ (*x* = 0, 0.01, 0.02, 0.03) samples after 30 cycles. Thus *τ*_*W*_ can be obtained from the linear fitting of Z_re_ *vs*. ω^−1/2^.

**Table 7:** The simulated data of the Li_1.20_[Mn_0.52−*x*_Zr_*x*_Ni_0.20_Co_0.08_]O_2_ (*x* = 0, 0.01, 0.02, 0.03) cathodes at 4.5 V from EIS spectra using the equivalent circuit shown in Fig. 11(e).

Sample	Cycle number	*R*_s_ (Ω)	*R*_*s*f_ (Ω)	*R*_ct_ (Ω)	Δ*R*_sf_ (Ω)
*x* = 0	1st	7.9	173.1	23.52	371.4
	30th	8.5	544.5	54.64	
*x* = 0.01	1st	7.5	158.7	22.05	287.1
	30th	7.4	445.8	45.23	
*x* = 0.02	1st	6.9	152.3	18.99	210.8
	30th	6.6	363.1	3487	
*x* = 0.03	1st	6.4	143.9	21.47	284.9
	30th	8.3	428.8	47.29

According to Eqs (1) and (2), after 30 cycles, with the Zr^4+^ doping contents increasing, the Zr^4+^-doped Li_1.20_[Mn_0.52_Ni_0.20_Co_0.08_]O_2_ samples exhibit the *D*_Li_^+^ values of 3.61 × 10^−14^ cm^2^ s^−1^, 8.32 × 10^−14^ cm^2^ s^−1^ and 5.46 × 10^−14^ cm^2^ s^−1^ respectively, higher than that (7.63 × 10^−15^ cm^2^ s^−1^) of the pristine electrode. Therefore, the Zr^4+^-doped Li_1.20_[Mn_0.52_Ni_0.20_Co_0.08_]O_2_ samples have demonstrated the superior rate capacity.

**Figure 13:**
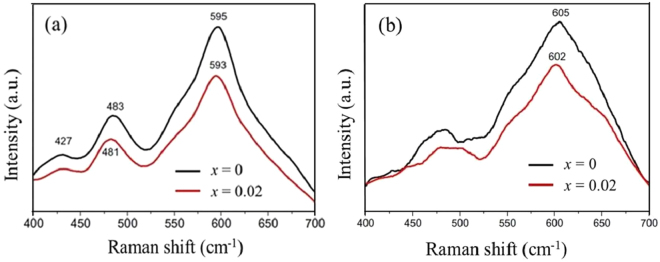
(**a**) Raman spectra of original Li_1.20_[Mn_0.52−*x*_Zr_*x*_Ni_0.20_Co_0.08_]O_2_ (*x* = 0, 0.02); (**b**) Raman spectra of cycled Li_1.20_[Mn_0.52−*x*_Zr_*x*_Ni_0.20_Co_0.08_]O_2_ (*x* = 0, 0.02) (100 cycles at 45 °C).

Figure 13 shows the Raman spectra of original and cycled Li_1.20_[Mn_0.52−*x*_Zr_*x*_Ni_0.20_Co_0.08_]O_2_ (*x* = 0, 0.02) (100 cycles at 45 °C). The Raman band at 427 cm^−1^ corresponds to the monoclinic Li_2_MnO_3_ phase, which can be observed in the spectrum of original Li_1.20_[Mn_0.52−*x*_Zr_*x*_Ni_0.20_Co_0.08_]O_2_ (*x* = 0, 0.02) in Fig. 13(a) and disappear in the cycled electrode in Fig. 13(b) owing to the disappearance of the monoclinic Li_2_MnO_3_ component after cycling^43^. Besides, the other two significant Raman bands at 483 and 595 cm^−1^ for the Li_1.20_[Mn_0.52_Ni_0.20_Co_0.08_]O_2_ (481 and 593 cm^−1^ for the Li_1.20_[Mn_0.50_Zr_0.02_Ni_0.20_Co_0.08_]O_2_ owing to the Zr^4+^ doping) belong to the bending E_g_ and stretching A_1g_ modes, respectively^44^ in Fig. 13(a). After 100 cycles, the Raman bands at 595 cm^−1^ for the Li_1.20_[Mn_0.52−*x*_Zr_*x*_Ni_0.20_Co_0.08_]O_2_ (*x* = 0, 0.02) have both shifted to higher values, indicating the cathode structure transformation from the layered to defect spinel structure^45^. The Raman band for the Li_1.20_[Mn_0.50_Zr_0.02_Ni_0.20_Co_0.08_]O_2_ have shifted from 593 to 602 cm^−1^ after 100 cycles, much lower than that of the pristine Li_1.20_[Mn_0.52_Ni_0.20_Co_0.08_]O_2_ (from 595 to 615 cm^−1^). Therefore, the Zr^4+^ doping have restrained the layered-to-spinel phase change of Li_1.20_[Mn_0.52_Ni_0.20_Co_0.08_]O_2_ during cycling, forming the superior electrochemical properties by Zr doping.

## Conclusions

In order to enhance the electrochemical properties of Li-excess Li_1.20_[Mn_0.52_Ni_0.20_Co_0.08_]O_2_, the different contents of Zr^4+^ have been doped into the pristine Li_1.20_[Mn_0.52_Ni_0.20_Co_0.08_]O_2_. After the Zr^4+^ doping, the cation mixing between Li^+^ and Ni^2+^ has been lowered and the cathode particles have been aggrandized. In comparison with the pristine cathode, the Zr^4+^-doped Li_1.20_[Mn_0.52_Ni_0.20_Co_0.08_]O_2_ samples have demonstrated the more stable cycling performance and higher rete capacities. Especially at high temperature (45 °C), the Zr^4+^ doping modification has delivered the more obvious superiority. After 100 cycles, with the Zr^4+^ doping contents increasing, the Zr^4+^-doped Li_1.20_[Mn_0.52_Ni_0.20_Co_0.08_]O_2_ samples exhibit the capacity retentions of 86.3%, 88.7% and 86.5% respectively, larger than that (82.8%) of the bare Li_1.20_[Mn_0.52_Ni_0.20_Co_0.08_]O_2_. The stronger total metal–oxygen bonding for the Zr^4+^-doped samples has mainly contributed to stabilizing the structure of cathode and improving the cycling performance. The Zr^4+^ doping modification has provided a potential approach to enhance the electrochemical properties of the Li-excess cathodes for Li-ion battery.
